# Variational Autoencoders-BasedSelf-Learning Model for Tumor Identification and Impact Analysis from 2-D MRI Images

**DOI:** 10.1155/2023/1566123

**Published:** 2023-01-17

**Authors:** Parvathaneni Naga Srinivasu, T. Balamurali Krishna, Shakeel Ahmed, Naif Almusallam, Fawaz Khaled Alarfaj, Nasser Allheeib

**Affiliations:** ^1^Department of Computer Science and Engineering, Prasad V Potluri Siddhartha Institute of Technology, Vijayawada, Andhra Pradesh 520007, India; ^2^Department of Computer Science and Engineering, Dhanekula Institute of Engineering and Technology, Vijayawada, Andhra Pradesh 521139, India; ^3^Department of Computer Science, College of Computer Sciences and Information Technology, King Faisal University, Al-Ahsa 31982, Saudi Arabia; ^4^Department of Management Information Systems, College of Business Administration, King Faisal University, Al-Ahsa 31982, Saudi Arabia; ^5^Department of Information Systems—College of Computer and Information Science, King Saud University, Riyadh, Saudi Arabia

## Abstract

Over the past few years, a tremendous change has occurred in computer-aided diagnosis (CAD) technology. The evolution of numerous medical imaging techniques has enhanced the accuracy of the preliminary analysis of several diseases. Magnetic resonance imaging (MRI) is a prevalent technology extensively used in evaluating the progress of the spread of malignant tissues or abnormalities in the human body. This article aims to automate a computationally efficient mechanism that can accurately identify the tumor from MRI images and can analyze the impact of the tumor. The proposed model is robust enough to classify the tumors with minimal training data. The generative variational autoencoder models are efficient in reconstructing the images identical to the original images, which are used in adequately training the model. The proposed self-learning algorithm can learn from the insights from the autogenerated images and the original images. Incorporating long short-term memory (LSTM) is faster processing of the high dimensional imaging data, making the radiologist's task and the practitioners more comfortable assessing the tumor's progress. Self-learning models need comparatively less data for the training, and the models are more resource efficient than the various state-of-art models. The efficiency of the proposed model has been assessed using various benchmark metrics, and the obtained results have exhibited an accuracy of 89.7%. The analysis of the progress of tumor growth is presented in the current study. The obtained accuracy is not pleasing in the healthcare domain, yet the model is reasonably fair in dealing with a smaller size dataset by making use of an image generation mechanism. The study would outline the role of an autoencoder in self-learning models. Future technologies may include sturdy feature engineering models and optimized activation functions that would yield a better result.

## 1. Introduction

Identifying the tumors and abnormalities in the human body through medical imaging for investigative analysis has become a regular practice. Various medical imaging technologies are used for diagnosis depending on the type of disease and the body part. The imaging must be performed for an early and precise abnormality diagnosis. The technologies include X-ray, which is used in the identification of bone fractures or for the identification of hard tissues [1], and computer tomography (CT) technology [2], which is widely used in contrasting various soft tissues such as the liver, lung tissues, and fat [3]. MRI technology is widely used in the diagnosis of abnormalities in neurology, cardiology, cancer, and soft tissue damage [4, 5], and a positron emission tomography (PET) scan is widely used in the detection of cancerous cells and malignant tissues [6].

**Figure 1:**
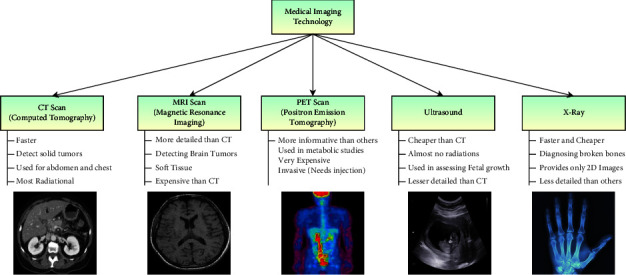
Image representing various imaging technologies.

MRI is most widely used in computer-aided diagnosis because of its low radiation and noninvasive nature. On the one hand, MRI can generate a more detailed report of soft tissues compared to CT scans. On the other hand, MRI technology can capture multiplaner images that can capture multiple angles in different planes. Moreover, MRI is capable of producing angiographic images without the use of any contrast material. The patient can be periodically investigated through MR imaging. The assessments of CAD models are being analyzed to assess the tumor's impact, which would assist in deciding the influence of the drug that acts on the abnormality. The current study identifies the tumor's progress as actual, enhanced, and whole tumors [7].  Figure 1 represents various medical imaging technologies that are used in smart diagnosis [8].

The existing state of art models that work over the segmentation procedures could only recognize the region of the tumor and they are not efficient in classifying the type of tumor and the progress of the tumor growth [9]. The other deep learning models like AlexNet [10], DenseNet [11], VGG-16 [12], Resnet-50 [13], and MobileNet [14] need tremendous training for attaining reasonable performance. There is a great demand for a model that works with minimal training data, especially to deal with novel diseases. Generally, the action of tumor recognition follows the same procedure as any other neural network model. The image undergoes multiple phases in tumor identification, including preprocessing the image to normalize the noise and enhancing the image's contrast for better recognition of the regions in the image. Then, the image is given as input to the algorithm to identify the features acting as a road map for the segmentation process, which is an interactive process. The proposed self-learning approach immediately performs image analysis by considering knowledge acquired from previous experimental studies and performs the automated segmentation of the MRI images. The integrated LSTM component is efficient in retaining parameters such as the learning rate of the model through weights and biases observed in previous epochs. Thereby optimizing the efforts for weight assessment and updating. The overall contributions of the current study are discussed as follows:

- The primary goal of the current study is to mechanize a self-learning model that can make a precise assessment of tumor progression with minimal data
- We discuss various existing tumor progression assessment models based on image segmentation and their limitations
- The autoencoders generate numerous images identical to the original MRI images for training
- Integrating the LSTM component with the self-learning model improves performance by maintaining adequate model parameters
- Using the grey-level co-occurrence matrix for the feature engineering task by the self-learning network model that assesses the number of occurrences of combinations of the pixels found in the input MRI image
- Analyzing the hyperparameters to determine the efficiency of the self-learning model
- Analyzing the performance of the proposed model against benchmark evaluation metrics like sensitivity, specificity, accuracy, and F1-score
- Analyzing the tumor impact through measures like tumor core, enhanced tumor, and whole tumor

The paper is structured as follows: the introduction presents the domain of the study and the author's contributions.  Section 2 offers the related work used in the automated segmentation of the MRI images to assess the tumor's progress.  Section 3 presents the proposed self-learning model.  Section 4 focuses on the experimental results and discussion part of the study. Finally, Section 5 offers the conclusions and future scope.

## 2. Literature Review

Various image segmentation approaches are available to localize the abnormality from the MRI images. The K-means-based segmentation algorithm is among the approaches that are most extensively used in semiautomated segmentation mechanisms that need the number of segments to be predetermined [15]. Moreover, it is simple to implement and requires less computational effort with limited iterations to segment the image. Unlike other segmentation approaches, it does not need training to perform segmentation. Still, it is limited to over, and under-segmentation of the images as the number of segments is predetermined. This approach is hardly used these days to segment medical images.

Fuzzy c-means [16] is the improvised version of the traditional K-means algorithm [17]. The pixels are allotted to the image segments based on the membership value rather than considering the distance measured in the K-means algorithm. However, this approach has a fixed number of segments in the image that fails to identify tiny regions significant in tumor identification. Moreover, the fuzzy C-means algorithm requires a high load for evaluating each pixel's membership to the corresponding segments. In a comparative analysis of various approaches for brain tumor identification from the MRI images [18], instructive fuzzy clustering means to have a misclassification error rate of 0.537%.

**Figure 2:**
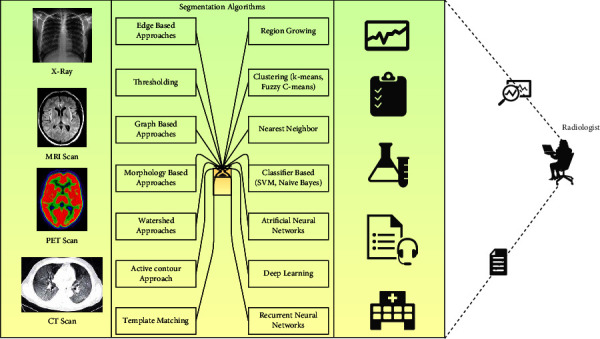
Image representing the various approaches to segmentation.

The MRI images are being segmented using fully automated mechanisms such as thresholding [19], region growing [20], and edge-based segmentation [21–23]. The threshold-based segmentation mechanism is suitable for MRI images, and the studies have proven that experimenting with diffusion-weighted MRI images has obtained 86% accuracy [24]. However, choosing the optimal threshold is challenging, and an inappropriate approximation may lead to poor segmentation. However, the region-growing mechanism has outperformed image segmentation by enhancing the sensitivity and specificity of recognizing malignant tissues. Nevertheless, the algorithm is susceptible to selecting the initial seed points [13]. In edge-based segmentation, the outputs are reasonably fair [25]. The image needs to be enhanced, and the edge-related information that needs to be highlighted needs rigorous preprocessing on the image, which requires more computational efforts.  Figure 2 represents the radiologist's various segmentation approaches in recognizing the abnormalities from the medical images.

The watershed-based image segmentation model works based on edge-based information that necessitates foreground and background planes as separated files that require high computational resources for preprocessing the image [26]. The watershed-based image segmentation model is well-systematized for handling homogeneous and heterogeneous malignant cases [4]. However, the watershed-based model fails to address the high-intensity images and noise. The atlas-based [27] approach works based on labeling and can efficiently handle the deformation model. Still, it is susceptible to the initial seed point, and the accuracy of the outcome depends on the topology.

The pixel label-based image segmentation [28] is a robust approach extensively used to segment medical images to recognize the abnormalities from the 2D MR images. Identifying the labeled data set of images would be challenging as the algorithm's accuracy relies on the machine's quality and quantity. Moreover, labeling the images for the training is not technically feasible, so the approach is hardly used in segmenting the medical-related images. Pixel label-based image segmentation has exhibited a mean accuracy of 80% on experimenting with the OASIS cross-sectional MR images, which is too low for sensitive medical data. Contour-based image segmentation [4, 29] is an improvised approach over pixel-labeling-based segmentation. It is among the most predominantly used image segmentation approaches to identify the objects and regions in an image. Although, the contour-based image segmentation approach fails to handle high-intensity images. Moreover, the images that have noise, such as the medical MR images, are susceptible to Gaussian and poison noise caused by thermal vibrations from the medical equipment or improper equipment calibration.

A probabilistic neural network (PNN) [30], in conjunction with learning vector quantization (LVQ), is efficient in segmenting the image in minimal computational time by optimizing the hidden layers. Self-organized Map (SOM) [31] model-based image segmentation is noticeable in differentiating the malignant tissues based on the image's spatial information. The SOM model needs excellent training, which is not always possible in unusual cases. Fuzzy recurrent neural network (FR-Net) [4] efficiently handles high-dimensional data like MR images. The FR-Net model works by fine-tuning the weights and biases over the parameters and the feedback loops that will assist in identifying the features that determine the abnormality of the image. FRNN needs an enormous computational effort to process the information, and the model needs tremendous training.

Artificial neural networks. (ANN) is a predominantly used segmentation approach for medical imaging technology, motivated by the biological neurons being practically implemented through artificial neurons controlled by the activation and transfer functions. Based on the underlying ground facts and working principles, the auto-encoders are designed in divergent architectures that can either adhere to the logic of a recurrent neural network (RNN) [32], which has obtained 93.82% on the implementation of 58 images obtained from the UCSB bio-segmentation benchmark dataset, and convolution neural networks (CNN) [33]. The ANN has the benefit of memorizing the entire network, and they have the competence to do with insufficient knowledge due to missing features for segmentation. Resultantly the ANN approaches are fault-tolerant and share parallel processing compatibility. The major limitation of the ANN approach is that the structure is determined for processing the data. It needs a tremendous computational effort to process the algorithm, and tracing the error in the network is challenging. Neural networks [1, 18] and model-based classification have exhibited better accuracy. Artificial Neural Networks performing the feature extraction over the dataset of size 428 MR images have exhibited a classification accuracy between 77–91% based on the size of the training set and the feature extraction strategy deployed.

Deep learning-based image segmentation is among the most widely used image segmentation models in recent years that would yield better accuracy in tumor identification [34]. The accuracy is 84% on experimenting over a BRATS-13 leaderboard set and 81% on a four-label test set on the BRATS-14 dataset. The abovementioned model needs a proportionately high-performance computer concerning the number of intermediate layers for segmentation. A hybrid approach with a combination of the pulse-coded neural network (PCNN) and feed-forward back neural network (FFBNN) [35, 36] technique identifies the preliminary seed points. The latter approach would setback the points that would stabilize the input, leading to an optimal image segmentation level. But the two algorithms need to be executed simultaneously, which requires more execution time and computational efforts and fails in handling unusual cases.  Table 1 presents the various existing approaches for tumor recognition.

## 3. Self-Learning Model with LSTM

The proposed SLNS-LSTM technique incorporates several components integrated for performing the automated segmentation and assessing the tumor's progress. The images are preprocessed internally to discard the noise, and then the images are segmented based on texture-based information. The information related to the previous experiment is loaded in a temporary memory, namely, the long short-term memory, which is widely used with recurrent neural networks. In image segmentation, feature selection is significant in attaining remarkable accuracy.

### 3.1. Self-Learning Model with Partial Training

The self-learning algorithm is technically efficient in addressing the segmentation of novel cases, and the algorithm will train itself to segment every case. Initially, the algorithm is partially trained with the available data set, and every time segmenting an image, the algorithm is self-trained. The algorithm will have an auxiliary memory to maintain the historical information of previous executions. The algorithm is sturdy to handle both noisy and noiseless images. The machine is trained with the noisy image at an acceptable level using the labeling approach [38] through a binary identifier to differentiate noise from noise-free images. The algorithm can differentiate various images while performing the automated segmentation of the images.

In automated segmentation for the abnormality diagnosis, it is crucial to distinguish brain tissues from nonbrain tissues. To be normalized, the original MRI image comprises much nonbrain tissue, such as the skull region, brainstem, thalamus, and brain fluid. The proposed algorithm is a layered approach for discarding all such nonbrain tissues for better statistical image analysis. An explicit algorithm is being executed in all the conventional mechanisms simultaneously to discard the nonbrain tissue that needs additional effort to perform the task. The self-learning component is among the multiple layers in the proposed algorithm, to which the outcomes of the previous executions are fed as the input. The progress of tumor growth is assessed by the area of the abnormality that has increased from the previous scan for a particular patient. The texture-based information is being considered in evaluating the progress of the tumor.

### 3.2. Variational Autoencoder for Image Generation

Dealing with the self-learning models, the models are desired to work with smaller datasets. On splitting the datasets as the training and testing partitions, the size of the data meant for training may not be adequate, resulting in the underfitting of the model. The variational autoencoders are used in the current study to generate images identical to the training images. The variational autoencoders consist of two components, namely, the encoder and the decoder. The encoder maps the original picture to a latent space and reconstructs the information in the latent space back into its initial dimensions performed by the decoder. The variational autoencoders (VAE) are probabilistic in assessing the process of feature assessment. VAE gives a probabilistic description of an observation in latent space. Thus, rather than developing an encoder that produces a single value to represent each latent state feature, it is preferable to use many values. VAE presents the probabilistic distribution for every attribute in the latent space. The probabilistic measure is associated with the joint model *p*_*α*_(*m*, *n*)=*p*_*α*_(*m*|*n*)*p*_*α*_(*n*) concerning the parameter *α* and posterior *q*_*β*_(*n*|*m*) concerning the parameter *β*. The prior images are used to sample the latent variable *n*, i.e., *n*  ~  *p*_*α*_(*n*), while the observation model is used to sample the observation variable *m*, i.e., *m* ~ *p*_*β*_(*m*|*n*). The optimized probability concerning the original images *x* is shown in the following equation:

(1) $$\begin{array}{c} p_{\alpha }\left(m\left|x\right.\right)=\int_{n}p_{\alpha }\left(m\left|n\right.,x\right)p\left(n\left|x\right.\right)dn\text{,} \end{array}$$

(2) $$\begin{array}{c} p_{\alpha }\left(m\left|n\right.,x\right)=\frac{p_{\alpha }\left(m\left|n\right.,x\right)p_{\alpha }\left(n\left|x\right.\right)}{p_{\alpha }\left(m\left|x\right.\right)}\text{.} \end{array}$$

Equation (2) corresponds to the posterior of the latent space, and as the variable *p*_*α*_(*m*|*x*) is accessible, the posterior is evaluated using the *q*_*β*_(*n*|*m*, *x*) ~ *p*_*α*_(*n*|*m*, *x*). The conditional evident lower bound is assessed using Jensen's inequality, as shown in the following equation:

(3) $$\begin{array}{c} L\left(m,x,\alpha ,\beta \right)=R_{q_{\beta }\left(n\left|m\right.,x\right)}\left[\log\frac{p_{\alpha }\left(m,n\left|x\right.\right)}{q_{\beta }\left(n\left|m\right.,x\right)}\right]\leq \log p_{\alpha }\left(m\left|x\right.\right)\text{.} \end{array}$$

The images generated using the variational autoencoders are used in training the self-learning network. The apparent loss function of VAEs, comprising a rebuilding component and a regularization element, may be precisely constructed using the statistical approach of variational inference; thus, the name variational autoencoders, is given a basic probabilistic model that represents the data.

### 3.3. Convolutional Operations in Self-Learning Network

**Figure 3:**
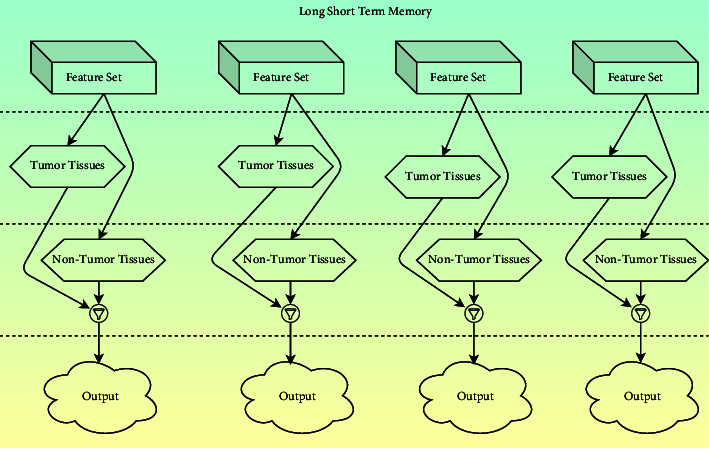
Block diagram of long- and short-term memory.

Long- and short-term memory has significantly impacted identifying the tumor from the MRI image. LSTM comprises two classes that are categorized as tumors and nontumor tissues. Whenever the image is fed as the machine's input, it classifies the tissues based on the antecedent experimental studies [39]. The proposed mechanism's LSTM component would be competent for learning abiding dependencies and environmental parameters for the past and future. Each layer in LSTM acquires the input from the first objective function of HARIS and generates the data for the second objective function by evaluating the feed-forward sequence $\overset{⟶}{ff}$ and feed-backward series that is being denoted by $\overset{´}{fb}$. The output sequence generated by the feature vectors is stated through equations (4) and (5). The block diagram of the LSTM module in the proposed approach is presented in Figure 3, which classifies the tumor tissues from the nontumor tissues associated with the feature set.

(4) $$\begin{array}{c} \overset{⟶}{ff}=\sigma \left(W_{i}\overset{⟶}{ff\;}{pix}_{i}+W_{\overset{⟶}{ff}}\;{\overset{⟶}{ff}}_{i-1}+{bias}_{\overset{⟶}{ff}}\right)\text{,} \end{array}$$

(5) $$\begin{array}{c} \overset{⃖}{fb}=\sigma \left(W_{i}\;\overset{⃖}{fb}{pix}_{i}+W_{\overset{⃖}{fb}}\;{\overset{⃖}{fb}}_{i-1}+{bias}_{\overset{⃖}{fb}}\right)\text{.} \end{array}$$

From the above equation, the variable *σ* represents the sigmoid function,  *W*_*i*_ denotes the weighted matrix, bias means the bias for both feed-forward represented by ${\;bias}_{\overset{⟶}{ff}}$ in equation (4) and feed-backward represented by ${\;bias}_{\overset{⃖}{fb}}$ in equation (5), and the variable pix_*t*_ represents the each input pixel sequence represented by pix = (pix1, pix2,…pixi).

Self-learning network-based segmentation (SLNS) is a layered approach comprising several convolutional layers that carry out the various crucial tasks in the procedure of automated segmentation that involves the preprocessing for the unwanted noise removal from the image using the adaptive fuzzy contourlet transforms [40]. Then, the resultant image is fed to the corresponding layers of the network to accomplish the image's automated segmentation by taking texture-related information into account. In each iteration, the feedback from LSTM is considered in differentiating the damaged tissues from nondamaged tissues [41].

SLNS approach-based MRI image segmentation requires partial training from various sources that involves acquiring the data related to the segmentation of the images from the labeled segmentation datasets and the knowledge that the algorithms have learned from the previous experimentation. In the proposed approach, every layer of the network is significant, one of the layers performs noise removal through adaptive fuzzy contourlet transforms, and the rest of the sublayers would evaluate the optimal number of segments and appropriate segment centroid, respectively, that are refined for several iterations until it reaches an optimal solution.

The original image is being processed by the noise removal layer of the proposed SLNS network using an adaptive fuzzy counter transform mechanism that enriches the image quality from magnitude and anisotropy perspectives [42, 43]. The idea of the adaptive contourlet transforms on various kernels that involve the Laplacian pyramid kernel and direction kernel over the image by preserving some of the critical information in the image, like edge-related information and minor regions in the medical images. Then the fuzzy contourlet transform works on the logic of selective enhancement of areas in the image, the Laplacian pyramid that classifies the single as low pass frequencies that are being decomposed that would lead to normalized signal strength and the high-pass frequencies that would enrich the sensitive and crucial points over the original MRI images. On the other hand, the Laplacian filter assesses the variance between the image and the resultant filtered image using a low pass filter that blurs the image to curb the low frequencies. The direction kernel would rebuild the images by enhancing the edge-related information by using the concept of frequency partitioning through the first-order derivative among the intensities of the pixels that are part of the same region that can be used in any direction.

#### 3.3.1. Integration of LSTM with SLNS Architecture

**Figure 4:**
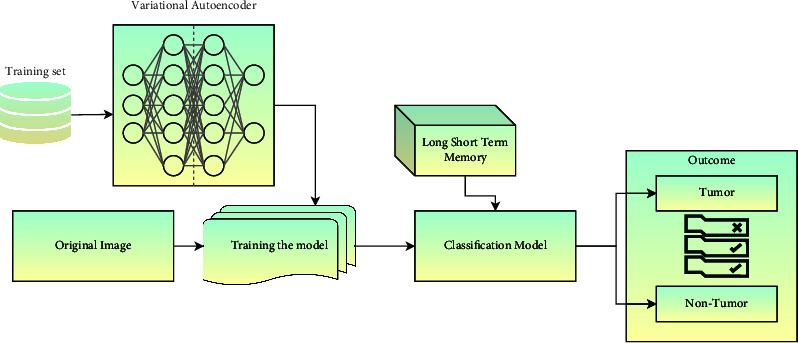
The block diagram of the proposed self-learning network model.

The LSTM memory module is integrated with the layered architecture of the self-learning network model, which holds the layers like the pooling layer and the convolution layers of the proposed architecture. The pooling layer's responsibilities are incorporated with the convolution layer that assists in incrementally decreasing the spatial dimensions for the depiction, minimizing the features needed for a thorough reorganization, and assessing the tumor from the MRI image. The convolution layer performs in decomposing the image to the maximum possible extent for approximating the tumor by using the properties that are being recognized. The convolution layer has a significant impact on the computation latency and the accuracy of the algorithm.  Figure 4 represents the architectural design of the proposed self-learning network model.

The technical tradeoff over the convolution layer is the down-sampling performed in the striding at the convolution layer. The more it decomposes, the more the accuracy of the classification. Still, there would be a considerable burden on the algorithm that can affect the computational latency of the model. In the proposed model, the LSTM component is integrated with the segmentation component of the proposed model; the LSTM model will maintain the information about the segmentation outcomes of the previous experimental instances. The LSTM and training set information are used in the classification process. The MRI images are then classified as tumor and nontumor images for diagnosing the tumor.

**Figure 5:**
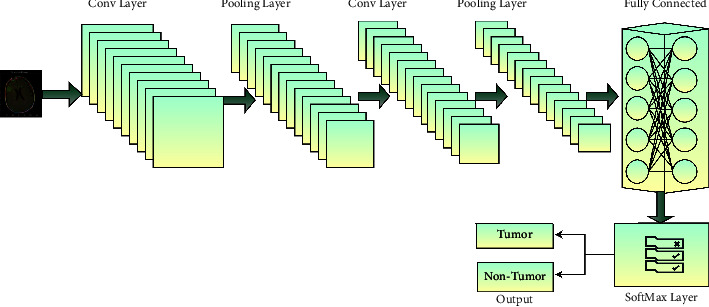
The layered framework of the self-learning model.

The self-learning model's layered approach comprises the convolution layer with the various kernels applied to the input MRI image to recognize the feature maps that will assist in the classification process. Then, the resultant outcome is processed using the kernel through the HARIS algorithm. The tensors of the second convolution layer are applied with the Max Pooling layer that down-sampling the input data for faster processing. The LSTM component handles the vanishing gradient problem of dealing with high-dimensional images. Incorporating the LSTM is a recognized dependency among the features in determining the abnormality regions in the segmentation process. The fully connected layer in the SNLS-LSTM architecture will map the features organized by the nonlinear associations in one layer with the activation unit in the upcoming layer. The SoftMax layer will assess the probabilistic measure of the image classified among the tumor and nontumor regions.  Figure 5 presents the layered framework of the proposed self-learning model.

### 3.4. Noise Normalization

The noise is predicted using the hybrid kernel to discard unwanted noise. The equation for noise analysis is as follows:

(6) $$\begin{array}{c} pix\left(i,j\right)={Gaussian}_{pix\_int}\left(i,j\right)-\left\{f_{z}\times 4\times \overset{2}{\underset{x=-2}{\sum }}\overset{2}{\underset{y=-2}{\sum }}\omega \left(m,n\right){Gaussian}_{pix\_int}\left(\frac{i-m}{2},\frac{j-n}{2}\right)\right\}\text{.} \end{array}$$

From equation (6), the variable  Gaussian_pix_int_ denotes the corresponding Gaussian kernel coefficient associated with coordinates (*i*, *j*), and the function *ω*(*m*, *n*) indicates standard deviation concerning the dimensional coordinates (*i*, *j*). The variable *f*_*z*_ is the fuzzier arbitrary value that is determined by the pixel membership following the image segment, whose value is defined as follows:

(7) $$\begin{array}{c} f_{z}\left({pix}_{i}\right)=\frac{1}{1+{\left|{pix}_{i}-t_{i^{\max}}/ki^{\max}\right|}^{2ui^{\max}}}\text{.} \end{array}$$

From the above equation, the variable *f*_*z*_(pix_*i*_) determines the pixel's fuzziness concerning the segment. and the variables *t*_*i*^max^_, *ki*^max^ and *ui*^max^ are the antecedent augments. pix_*i*_ is the ith pixel in the input image.

### 3.5. Feature Selection for Segmentation

Feature selection is one of the pivotal phases of image segmentation, as the performance of the segmentation algorithm depends on the features through which the image is being segmented. For feature selection, the grey-level co-occurrence matrix (GLCM) is very suitable for identifying the features from the MRI images [3]. GLCM is a statistical texture-based approach that works on how frequently the pixel with the corresponding intensity has occurred in the considered image. For each pixel considered (*p*, *q*), the key value represents the frequency of occurrence horizontally adjacent to the corresponding pixel intensity *p*. If an image of 228 bits is considered, the GLCM matrix would be 228 × 228. The coefficient of the GCLM for the square size image is approximated through the following equation for an MRI image with “*p*” rows and “*q*” columns:

(8) $$\begin{array}{c} GCLM\left(x,y\right)=\frac{GCLM\left(x,y\right)}{\overset{p}{\underset{x=1}{\sum }}\overset{q}{\underset{y=1}{\sum }}GCLM\left(x,y\right)}\text{.} \end{array}$$

By conducting normalization on the coefficients of GLCM, they are represented by probability instead of frequency of occurrences. The terms are further divided by the entire list of possible combinations. The same has been presented in the following equation with a displacement vector (*d*_*p*_, *d*_*q*_):

(9) $$\begin{array}{c} {GCLM}_{n}\left(x,y\right)=\overset{m}{\underset{x=1}{\sum }}\overset{n}{\underset{y=1}{\sum }}\left\{\begin{array}{c} 1,if\;I\left(x,y\right)=1\;,\\ \;I\left(p+d_{p},q+d_{q}\right)=0 \end{array}\right\};\;else\;0\;otherwise\text{.} \end{array}$$

### 3.6. Pixel Fitness Evaluation

Upon processing the input image to discard the unwanted noise, the image is fed as input to the proposed segmentation mechanisms associate layer concerning the pixels' intensity with a general assumption that the image would be 23 from the previous experiences. The fitness of appropriate segments is computed through the following equation:

(10) $$\begin{array}{c} {fitness}_{fun}=\left(p\times \frac{{Total}_{img\_pix}}{{total}_{seg}}\right)+\left(q\times \frac{{Total}_{sp}}{{pix}_{seg}}\right)\text{.} \end{array}$$

In the above equation, the variables *p* and *q* are the arbitrary coefficients associated with the accuracy, and the efficiency of the fitness function *w*, *p* determines the inter-class variance that must be maximally assessed through equation (11). The variable *q* determines the intra-class correlation that must be at its maximum, which is assessed using equation (10). Either of those will be the sublayers of the segmentation layer of the proposed model. The variable  Total_img_pix_ designates the sum of pixels in the entire image, total_seg_ designates the sum of image segments and  Total_sp_ denotes the sum of seed points in the considered image and pix_seg_ denotes the total number of pixels concerning the segment “seg.” The interclass variance is assessed as follows:

(11) $$\begin{array}{c} \sigma^{2}{inter}_{cls}\left(\theta \right)=\sigma^{2}total\left({cls}_{\theta }\right)-\sigma^{2}p_{\mathrm{int}\left(C_{\theta }\right)}\text{,} \end{array}$$

(12) $$\begin{array}{c} \sigma^{2}{inter}_{cls}\left(C_{\theta }\right)=\overset{C_{\theta }-1}{\underset{i=0}{\sum }}p\left(i\right)\overset{\max\;-1}{\underset{i=C_{\theta }}{\sum }}p\left(i\right){\left[\mu_{1}\left(C_{\theta }\right)-\mu_{2}\left(C_{\theta }\right)\right]}^{2}. \end{array}$$

Equation (11) is expanded as equation (12), and the class threshold is denoted by *C*_*θ*_ that is approximated using the concept of the fuzzy entropy-based thresholding (FET) mechanism. The arbitrary variables *μ*_1_, *μ*_2_ The above equation would approximate the average intensities of the pixels in the corresponding segment.

A fuzzy entropy-based thresholding mechanism would assess the pixel's correlation concerning a segment in the image that tells to what degree the pixel belongs to the region. The fuzzy entropy threshold is determined using Shannon's entropy, whose values would always like within the range of 0 and 255 for any given grayscale image defined in equation (13), the variables *μ*_tumor_cls__, *μ*_non_tumor_cls__ It would denote the evaluated fuzzy membership concerning the image segment corresponding to the tumor and nontumor classes.

(13) $$\begin{array}{c} \theta =\overset{\max}{\underset{i=1}{\sum }}\mu_{{tumor}_{cls}}\left(i\right)\log_{2}\mu_{{tumor}_{cls}}\left(i\right)-\overset{255}{\underset{i=\max\;+1}{\sum }}\mu_{{non\_tumor}_{cls}}\left(i\right)\log_{2}\mu_{{non\_tumor}_{cls}}\left(i\right)\text{.} \end{array}$$

The value assessed for the intraclass correlation, as stated in equation (14), would determine how closely the pixels with the same segment are related to each other. It is desired to have higher values for strongly correlated pixels within the same segment. The standard deviation assesses the correlation between the pixels and the segment.

(14) $$\begin{array}{c} I_{correlation}=\frac{\sigma_{seg}^{2}}{\sigma_{seg}^{2}+\sigma_{img}^{2}}\text{.} \end{array}$$

From equation (14), the component *σ*_seg_ denotes the standard deviation among the pixels concerning the corresponding segment computed locally, and the component *σ*_img_ indicates the standard deviation of the entire image that is calculated globally.

In working with Long- and short-term memory, the brain tissues are classified as tumors. Nontumor tissue classes from the previous execution and the classes are assigned weights based on the equations (15) and (16) that are stated below for each sample *I*_*i*_, which are assessed following the likelihood ratio of either class of tissues identified from 2D MR images of tumor(*i*)/nontumor(*i*).

(15) $$\begin{array}{c} W_{p}\left[\frac{tumor\left(i\right)}{nontumor\left(i\right)}\right]=\int_{i=0}^{n}f\left(i\right)\frac{nontumor\left(i\right)}{nontumor\left(i\right)}tumor\left(i\right)dx\text{,} \end{array}$$

(16) $$\begin{array}{c} \int_{i=0}^{n}f\left(i\right)tumor\left(i\right)dx=W_{q}\left[f\left(i\right)\right]\text{.} \end{array}$$

### 3.7. Data Collection

The labeled data for the partial training of the self-learning model is obtained from the BRATS 2015 [44] open-source dataset consisting of 220 severely affected patients' MRI image data and 54 acutely affected patient data. The dataset consists of T1-weighted, T1c-weighted, T2-weighted, and FLAIR-weighted MRI images labeled with the ground facts associated with the tumor. For partial training of the model, two parts of the dataset are used for training, and eight parts are considered for validation out of ten parts of the overall dataset. The image samples were downsized to 228 × 228 for the experimental study. We have collected 736 random patches at periodic intervals to check the development of classification performance during training, with equal numbers taken from all of the validation images in the dataset. To approximate the real proportion of tumors and healthy tissue, patches are evenly selected from the brain area. The benchmark dataset is considered over the real-time dataset in the current study, as the benchmark datasets are standardized and formatted to maintain uniformity across the samples, which would yield a better training performance.

## 4. Localization of the Tumor Region

The heuristic approach of real-time image segmentation (HARIS) has been incorporated for better performance and accuracy [45]. The algorithm would segment every consecutive experimentation concerning its previous experiment and the available training data. LSTM would support storing the state information in determining the tumor and non

tumor regions. The working procedure of the HARIS algorithm is presented in this section, and the libraries and environment settings used in the real-time implementation of the mechanism are presented.

### 4.1. HARIS Algorithm

The heuristic approach for real-time image segmentation technique is a multiobjective, function-based automated image segmentation algorithm that can automatically segment an image with reasonable computational efforts compared to its counterparts. The initial object function segments the medical MRI image, and the later objective function refines the segmented image, which continues for a few iterations until the stopping criteria are reached. Initially, the algorithm approximates the number of segments at the beginning through the elbow approach rather than choosing random segments like any other approach. The initial count of segments is determined through the following equation:

(17) $$\begin{array}{c} {initial}_{seg}=\overset{j}{\underset{{seg}_{i=1}}{\sum }}\underset{{pix}_{n}\in t_{i}}{\sum }\left|\left|{pix}_{n}-{centroid}_{i}\right|\right|\text{.} \end{array}$$

As mentioned earlier, equation (15) is executed recurrently until there is no significant change in the variable's value initial_seg_ Based on the ground facts, the value approximates 23 in the beginning iteration. In equation (17), the variable *j* pertains to the approximated number of segments in the previous iteration, and for the first iteration, the value of *j* would be 23. The variable seg_*i*_ designates the *i*^th^ a segment of the image whose range of values would be between 1 and the maximum limit denoted by the variable *j*. The variable pix_*n*_ designates the sum of the pixels in the nth segment. The variable centroid_*i*_ illustrates the approximated centroid of the *i*^th^ segment.

### 4.2. Identification of the Segment Centroid

The process of choosing the centroid of the segment is pivotal in the process of an optimal level of segmentation of the MRI image, as all the pixels around the centroid would be assigned to the corresponding centroid concerning some criteria that can be either a distance measure or the fitness function for assigning the pixel. The appropriate segment centroid is approximated through the following equation:

(18) $$\begin{array}{c} {seg}_{centroid}=\overset{\max}{\underset{pix=1}{\sum }}\sum_{segment=1}^{n}{fit}_{pix,seg}di\;st\left(x_{pix},{centroid}_{seg}\right)\text{.} \end{array}$$

From the above equation (14), seg_centroid_ designates the recognized latest segment centroid of the segment seg, and the variable fit_pix,seg_ designates the approximated fitness of the pixel pix concerning the segment seg. centroid_seg_ denotes the centroid of the image segment seg, and the variable *x*_pix_ is the *x*^*th*^ pixel of the segment. di st(*x*_pix_, centroid_seg_) designates the distance measured between the pixel and the corresponding image segment. In assessing the distance measure, the Mahalanobis distance measure is used to assess the mean of the feature vector, which is approximated from the mean value of intensities in the corresponding segment. The Mahalanobis distance *d*^2^(int, pix_*n*_) is assessed for the pixel pix_*n*_ with the intensity, int is approximated from the following equation:

(19) $$\begin{array}{c} d^{2}\left(\mathrm{int},{pix}_{n}\right)=\left(\mathrm{int}-\mu^{i}\right)\mathrm{int}D_{x}^{-1}\left(\mathrm{int}-\mu^{i}\right)\text{.} \end{array}$$

In the above equation, int*D*_*x*_^−1^ denotes the inverse of the matrix that has been produced from the covariance matrix in the following equation:

(20) $$\begin{array}{c} \mathrm{int}D_{x}^{-1}=\left(x_{i}-1\right)S_{n}^{-1}\text{.} \end{array}$$

The individual in each segment with concern to the segment centroid is determined from the following equation:

(21) $$\begin{array}{c} S_{n}=\overset{\max}{\underset{i=1}{\sum }}\left({\mathrm{int}}_{i}^{\max}-\mu_{\max}\right){\left({\mathrm{int}}_{i}^{\max}-\mu_{\max}\right)}^{T}\text{.} \end{array}$$

#### 4.2.1. Objective Function-I

The objective function-I performs the image's task by approximating the random segments through the elbow approach and assigning the pixels to it by evaluating the membership of the pixel to the corresponding segment. The pixel likelihood to the corresponding segmentation is assessed concerning the centroid of the segment that is evaluated from the following equation:

(22) $$\begin{array}{c} C_{r}=\frac{1/I_{C_{r}}\left(I_{1}^{2}+I_{2}^{2}+\ldots +I_{\max}^{2}\right)}{1/I_{C_{r}}\left(I_{1}+I_{2}+\ldots +I_{\max}\right)}\text{.} \end{array}$$

In the above equation, the corresponding segment centroid is identified by the variable *C*_*r*_, and the range of *r* lies between 1 and max. The objective function for estimating the pixel allotment is evaluated using the following equation:

(23) $$\begin{array}{c} f\left(x\right)=\left(\alpha \times \frac{{tot}_{pix}}{{pix}_{r}}\right)+\left(\beta \times \frac{{tot}_{cent}}{{pix}_{s}}\right)\text{.} \end{array}$$

In the above equation, the variables *α*, *β* are the two arbitrary variables that decide the accuracy and performance of the HARIS algorithm. The variable *α* determines the interclass variance, and the variable *β* determines the intraclass correlation of the pixels concerning the centroid of the segment. tot_pix_ designates the sum of pixels, pix_*r*_ denotes the sum of pixels in the region *r* of the image and tot_cent_ designates the sum of segments in the image and pix_*s*_ designates the number of pixels in the region s. The values of two arbitrary variables that are illustrated as the deciding factors for the performance of the algorithm are defined through the following equations:

(24) $$\begin{array}{c} \alpha =\frac{\sigma_{i}^{2}}{\left(\sigma_{i}^{2}+\sigma_{e}^{2}/2\right)}\text{,} \end{array}$$

(25) $$\begin{array}{c} \beta =\overset{\max}{\underset{C_{r}=1}{\sum }}\omega_{r}\left(x\right)\sigma_{r}^{2}\left(x\right)\text{.} \end{array}$$

In the above equation (22), the variable *α* determines the intraclass correlation, which figures out how closely the pixels are close to each other in concern to the centroid of the pixel, and the variable *β* in equation (23) determines how dissimilar the pixels are among the segments were the interclass variance designates that. The fitness is now being evaluated to decide whether to increase or decrease the number of segments. The fitness is evaluated through equation (25), and the algorithm for the HARIS algorithm is shown in Table 2.

(26) $$\begin{array}{c} f\left(C_{i}\right)=1+abs\left(f\left(x\right)\right)if\;the\;value\;of\;f\left(C_{i}\right)<0,\;a\;new\;segment\;is\;to\;be\;add\;e\;d\text{,}\\ f\left(C_{i}\right)=\frac{1}{1+f\left(x\right)}if\;the\;value\;of\;f\left(C_{i}\right)>0,the\;segment\;needs\;to\;be\;redu\;ced\text{.} \end{array}$$

#### 4.2.2. Objective Function-II

Objective function-II is to fine-tune the resultant outcome of objective function-I. The major focus of the current objective function is to identify the optimal segment centroid of the global best solution. All the membership function assigns all the pixels corresponding to the segment centroid based on the objection function and is assessed through the following equation:

(27) $$\begin{array}{c} {sp}_{i}=rand\left(0,1\right)\times {fitness\_sp}_{i-1}+rand\left(0,1\right)\times \left({GB}_{{sp}_{fitness}}-{fitnesss_{sp}}_{i-1}\right)\text{.} \end{array}$$

From the above equation, sp_*i*_ designates the current evaluation of the fitness of the seed point of the segment, and the variable fitness_sp_*i*−1_ designates the fitness of the seed point in the previous iteration.  GB_sp_fitness__ denotes the segment seed point's fitness value considered the global best in fitness, and the values are assessed iteratively.

### 4.3. Specifications and Environment

The following is the environment set up for the practical implementation of the algorithm. The experimentation has been conducted on several input images for a precise assessment of the accuracy of the proposed system.  Table 3 specifies the platform and environment where the proposed model has been implemented.

## 5. Experimental Results and Analysis

The SLNS-LSTM technique has been executed over the medical MRI images obtained from online repositories. Moreover, the experimentation is performed on MRI images of variable size. It is observed that the algorithm has exhibited better performance for smaller-size images than other algorithms. The self-learning approach is being evaluated through various benchmarks, including sensitivity, specificity, accuracy, and F1- Score. On the one hand, the experimental values of true positive values determine how well the self-learning approach correctly recognizes the abnormality.

Moreover, the true negative determines how well the proposed method accurately identifies the nontumor areas in the human brain. The false-positive rate determines how often the proposed approach fails to recognize the tumor region correctly. Furthermore, the false-negative rate determines how often the proposed method fails to recognize the nontumor region correctly. Based on TP, TN, FP, and FN values are used to assess the values on the experimentation of the proposed approach for several rounds, and the accuracy is assessed accordingly.

**Figure 6:**
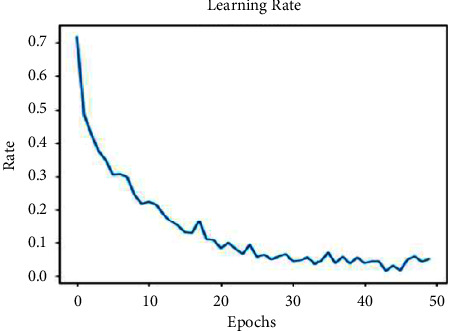
Image presenting the learning rate of the model.

The proposed self-learning model is initially trained with 20% of the dataset with the labeled data, and the model is designed to learn from the experimental outcome consistently. The SLNS model would exhibit higher learning factors at initial epochs and lesser at later stages as the model would have gained sufficient knowledge for abnormality identification. The learning rate of the model is presented in Figure 6 over 50 epochs. It can be observed that the model has attained a reasonable learning factor of 0.08 at 38 epochs. However, the learning rate is further reduced later in experimentation by accruing the knowledge from experimental studies.

**Figure 7:**
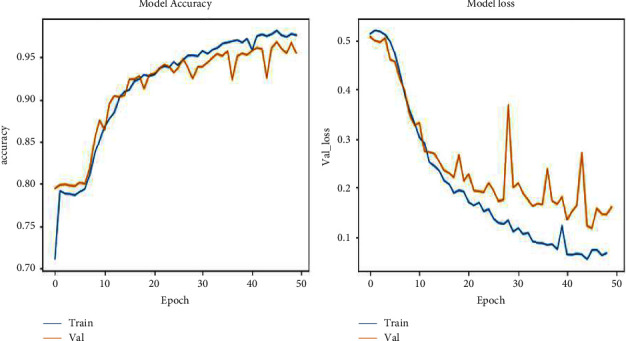
Graphs present the hyperparameters of the proposed model.

The other hyperparameters, such as the training and validation loss and the training and validation accuracies, determine the model's performance concerning the underfitting and overfitting situations in training the model. Underfitting happens when a machine does not learn from the data and does not generalize effectively across the validation data. The training accuracy plot could be flatter or have significant loss values, indicating that the model did not learn the training samples. Overfitting occurs when a model has learned excessively from the training samples, resulting in unpredictability while evaluating the validation data. The training loss plot falls as the number of epochs increases, and the validating loss plot drops to a level before rising. The corresponding graphs of loss and accuracy measures are shown in Figure 7.

**Figure 8:**
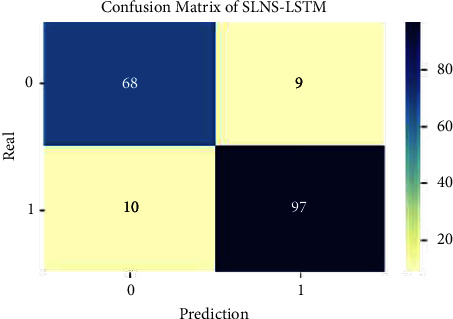
Confusion matrix of the proposed model.

The experimental outcome of the SLNS-LSTM techniques is compared with its counterparts, and the tabulated values are presented in Table 4. The performance of the SLNS with the LSTM algorithm is superior to that of the SLNS alone. Furthermore, there is a noticeable improvement in accuracy with almost the same level of training. The confusion matrix of the current study is presented in Figure 8.

The computational latency of the proposed approach is assessed through the execution time. The proposed approach has better performance when compared to others. However, there is a technical tradeoff, such as execution time. The execution time of the proposed approach is slightly more than that of the HARIS algorithm. Nevertheless, the proposed approach has exhibited better accuracy for MRI images. The impact of the tumor is assessed at the tumor core, whole tumor, and enhanced tumor to identify the tumor's progress in treating the patients. Consequently, the proposed approach would effectively identify the tumor's growth and help provide better treatment to the patients. The enhanced tumor in the tumor region is suspected to be spread compared to the earlier diagnosis of the ailment of the corresponding patient.

**Figure 9:**
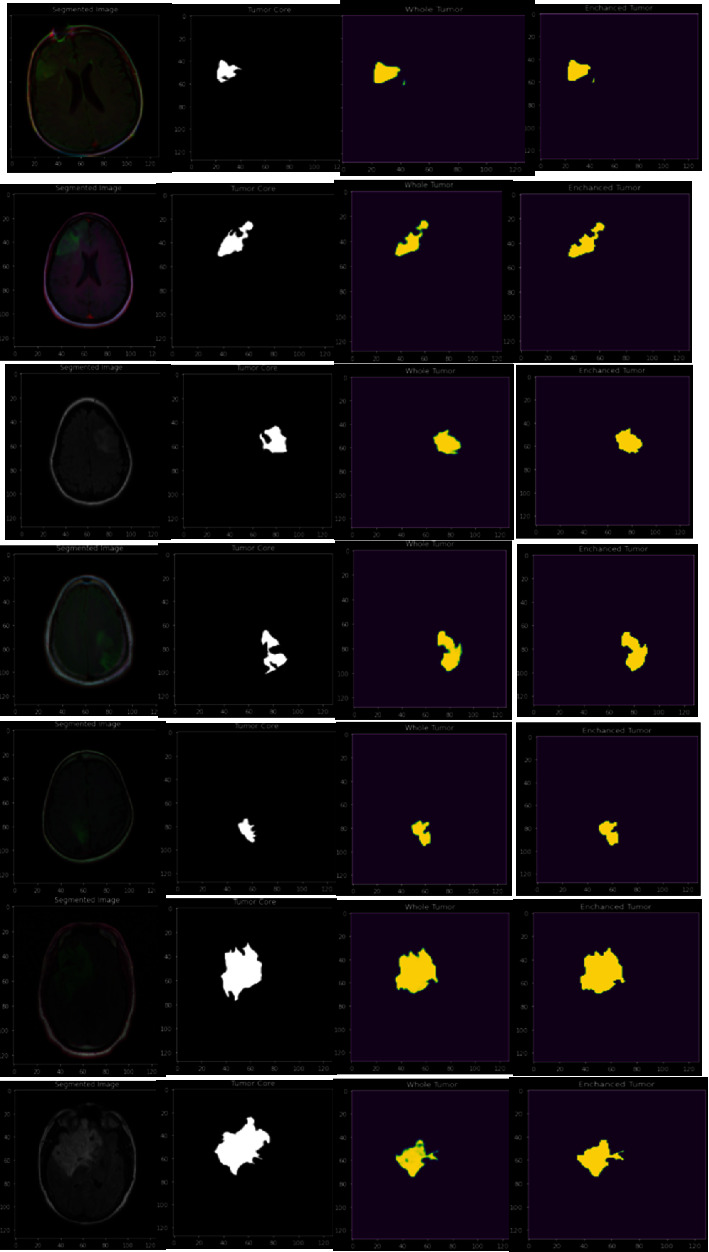
Segmented MRI images as the outcome of the proposed model.

Figure 9 represents the outcomes at each stage of the proposed algorithm's execution over the Jupiter notebook 5.7 software. The leftmost image represents the original image. The second image from the left denotes the segmented image representing the tumor core. The third image is the approximate whole tumor. The fourth image denotes the enhanced tumor whose area is larger than the tumor core. The texture-based information is significant in determining the impact of the tumor and the enhanced tumor region from the 2D MRI image.

Table 5 presents the analysis of the tumor's impact concerning the texture of the tumor region. The outcome is also highlighted by applying the colormap to the resultant effect based on the tumor region's texture values. Tumor Core designates the actual tumor region where the impact is significantly observed. The enhanced tumor designates the less impacted region based on texture-based information. The whole tumor designates the entire tumor region from the MRI image. The values might have deviated from the ground facts, and the erroneous index has been indicated along with each computed damaged index [48].

With the change in food habits and environmental conditions, there are multiple different diseases that human beings are undergoing. Consequently, new methods that include artificial intelligence and robust algorithms can work with minimal training to support medical decision-making. Moreover, approaches such as convolution neural networks and fuzzy recurrent neural networks need tremendous training in the existing cases to attain better accuracy. On the one hand, the proposed method productive can recognize the abnormality with high precision with the knowledge acquired from self-learning from the previous cases. The algorithm would keep on enriching the performance after that. In the current study, various performance contribution techniques like dropout factor and batch normalization techniques are not performed in the current study. The hyperparameters finetuning would also assist in better performance of the model. The feature selection techniques like gray-level co-occurrence matrix might be used in future models as feature engineering would have a significant impact on the performance of the model. The current models lack the above-discussed performance optimization mechanism that has resulted in an accuracy of 87.9%, which could be further improved by fine-tuning the same.

On the other hand, the self-learning algorithm relies on the HARIS algorithm for automated image segmentation over multiple iterations. Moreover, long- and short-term memory is used with the recurrent neural network for handling and preserving the state information of irrational long-term dependencies for predicting the time series data. In this experiment, the proposed approach has outperformed when compared with other existing self-learning-based algorithms. By incorporating the LSTM-based approach, the algorithm would perform much faster in assessing the tumors' progress and have better accuracy in differentiating the tumors and nontumor regions compared to its counterparts.

## 6. Conclusions

The current research examines the role of long- and short-term memory in improving the efficiency of self-learning network-based classification by feeding the network state information from an MRI picture. The insights from the pictures produced by the variational autoencoder are used as input for the tumor model's differential categorization. Furthermore, the self-learning technique can identify tumor progression with less training, resulting in quicker execution than its competitors. Self-learning models use less computer resources since they require minimum training and make the model acceptable for tumor diagnosis. The experimental results showed a decent accuracy of 89.7%. The proposed approach has outperformed other machine learning models' accuracy and execution time. The current study is confined to a limited number of samples for the training and testing purposes. The images are generated by the variational autoencoder for the training purpose. The proposed model has yielded an acceptable performance, but by optimizing the hyperparameters, the accuracy of the model can be further enhanced.

The SLNS model's performance has been greatly improved by including the LSTM module. However, the suggested model's performance may be improved further by using bi-directional LSTM and self-labeling techniques. Optimizing the class weights at each iteration will result in a promising result that may be explored for future perspectives. The deep variational autoencoder produces images of comparable quality to the variational autoencoder alone. The deep variational autoencoder, on the other hand, requires more computer resources than the other standard techniques. This future direction would assist in building a robust model that could efficiently detect the progress of the brain tumor. The earlier analysis of the tumor impact would assist in providing the appropriate treatment and decision-making.

## Figures and Tables

**Table 1:** Summary of various tumor recognition techniques.

Approach	Outcome	Limitations
K-means [37]	K-means for classification alongside average precision and intersection over union approaches for object reorganization in real-time images and splitting up the objects in the image	The approach is susceptible to oversegmentation or undersegmentation as the *k* value is fixed. A larger *k* value would result in oversegmentation, and a smaller *k* value would result in undersegmentation
Thresholding [38]	The author has experimented with the thresholding approach over diffusion-weighted MRI images for lesion identification using Gamma law transformation compared to contrast stretching	At most, care must be taken while picking the optimal threshold value. Wrongly choosing the threshold value may lead to inappropriate segmentation
Region growing [20]	Multispectral MRI image segmentation has been performed for segmentation and has exhibited better accuracy working with noisy MRI images. Fuzzy knowledge-based region growing has performed better than the traditional area growing algorithm	The resultant output image is susceptible to the initial seed point. There are chances where the algorithm might end up in global maxima or global minima
Edge-based segmentation [23, 25]	The authors have proposed working with Sobel edge detection to retain information that assists in the exact localization of the tumor through laser therapy	The image has to be preprocessed rigorously so that the edge-related information is elaborated, which would incur a high computational effort
Watershed [26]	Watershed-based segmentation is performed with different combinations of features that can effectively localize the tumor from the MRI	Considerable efforts must be needed during the preprocessing as the fore region is separated from the background region
Pixel-label-based image segmentation [28]	Pixel-label-based segmentation through encoder and decoder networks of SegNet layered architecture for image segmentation. It is observed with some enhancements. The outcome is very much pleasing, like natural outdoor images	The availability of the labeled training set for unusual tumor types is a challenging task. The segmentation approach is not suitable for assessing the progress of the tumor
Probabilistic neural network [30]	LVQ- based PNN system classification is better in the MRI image classification, with a minimal processing time of 79% less than conventional PNN	This approach needs considerable training for better accurate results, which cannot be used in unusual cases without sufficient training-set data
Fuzzy recurrent neural networks [37]	FR-net model is used to segment the high-dimensional MRI images with an accuracy of 87.8% and comparatively faster than various supervisory models	FR-net model needs considerable computational efforts and tremendous training to obtain reasonable accuracy
Artificial neural networks [32, 33]	RACE-net architecture efficiently addresses overfitting by adjusting the dependencies and CNN through a small kernel that assists in implementing the more profound architecture that also addresses overfitting by assigning fewer weights	ANN can be efficiently implemented through CNN and RNN. But either of those approaches needs tremendous training, which would significantly impact the computational latency, and a high-performing machine is required for implementation

**Table 2:** Algorithm for image segmentation using the HARIS technique.

Algorithm: HARIS algorithm for image segmentation
**Start**
Set the value of “max”
//Determine the centroid of the segment through, *S*_*n*_=∑_*i*=1_^max^(int_*i*_^max^ − *μ*_max_)(int_*i*_^max^ − *μ*_max_)^*T*^
//Pixel assignment based on the Mahalanobis distance *d*^2^(int, *pix*_*n*_) stated as follows: *d*^2^(int, *pix*_*n*_)=(int − *μ*^*i*^)int*D*_*x*_^−1^(int − *μ*^*i*^)
//Assess the intraclass correlation determined by *α*, $\alpha =\frac{\sigma_{i}^{2}}{\left(\sigma_{i}^{2}+\sigma_{e}^{2}/2\right)}$
//Assess the interclass variance determined by *β*, by assuming *β*=0 initially
for the value i = 0 to max-1, *β*=∑_*C*_*r*_=1_^max^*ω*_*r*_(*x*)*σ*_*r*_^2^(*x*)
end of for
//Implementing the first objective function-I
set the value of parameters like tot_pix_, pix_r_, tot_cent_, pix_s_, *f*(*x*)=(*α* × *tot*_*pix*_/*pix*_*r*_)+(*β* × *tot*_*cent*_/*pix*_*s*_)
return the value of f(x)
//Identify the fitness_sp_*i*_, *GB*_sp_fitness__ from the previous iteration
Assume *max* represents the sum of segments in the image
for the value i = 0 to max-1, sp_*i*_=*rand*(0,1) × *fitness*_*sp*_*i*−1_+*rand*(0,1) × (*GB*_*sp*_*fitness*__ − *fitness*_*sp*__*i*−1_), *seg* = *evaluate* (*α*, *β*)
return the value of *sp*_*i*_ and *seg*
end of for
End

**Table 3:** Table representing the system specifications.

Environment aspects	Specification
Machine	HP pavilion series
Processor	Core i7-10510U quad core
Architecture	64-bit
Memory	16 GB DDR4-SDRAM
Graphics	NVIDIA GeForce MX250
Operating System	Windows 10
Platform	Python
Tool	Jupiter notebook
Libraries	CV2, Matplotlib, pyplot, NumPy, pandas, torch

**Table 4:** The performances of various approaches.

	Number of samples	Sensitivity	Specificity	Accuracy	F1-score	Execution time (s)
HARIS [22]	1490	0.789	0.842	0.751	—	324
CNN [25]	1490	0.795	0.854	0.760	—	975
SLNS [25]	736	0.828	0.879	0.799	—	782
KNN [46]	700	0.460	0.500	0.780	—	—
GoogleNet [46]	700	0.848	0.960	0.896	—	—
VGG-16 [46]	700	0.812	0.884	0.844	—	—
AlexNet [46]	700	0.843	0.923	0.876	—	—
ResNet-50 [47]	253	0.930	—	0.890	0.900	—
ResNet-101 [47]	253	0.740	—	0.740	0.730	—
Inception-V3 [47]	253	0.710	—	0.750	0.740	—
SLNS with LSTM	736	0.871	0.915	0.897	0.877	455

**Table 5:** Impact of the brain tumor concerning various approaches.

	Tumor core	Whole tumor	Enhanced tumor
HARIS	2.87221	3.88981	3.011942
CNN	2.77638	3.79623	3.110214
SLNS	2.86786	3.80223	3.082932
SLNS with LSTM	2.89383	3.87081	3.105427

## Data Availability

No data were provided for the current study.
